# Alcohol Use Trajectories Across 24 Years and Future Dementia Risk: Evidence from the Health and Retirement Study

**DOI:** 10.1093/geroni/igaf122.4067

**Published:** 2025-12-31

**Authors:** Alonzo Mendoza, Carlos Araujo-Menendez, Armando Lemus, Ariana Stickel

**Affiliations:** San Diego State University, San Diego, California, United States; San Diego State University/University of California, San Diego, San Diego, California, United States; San Diego State University, San Diego, California, United States; San Diego State University, San Diego, California, United States

## Abstract

We aimed to determine alcohol use trajectories and their relation to future dementia risk. Participants included 3,488 adults from the Health and Retirement Study (HRS; waves 1992-2016) who underwent a dementia evaluation (cognitively intact, mild cognitive impairment, and dementia) as part of the 2016 Harmonized Cognitive Assessment Protocol (HCAP) and self-reported alcohol consumption during at least two study visits. Alcohol consumption composite scores (number of days drinking, drinks per day, and number of binges in the past 3 months) were used to model trajectories using latent class mixed models (LCMM). The number of latent classes was determined based on fit indices (AIC, BIC, log-likelihood, entropy) and the interpretability of trajectories. Multinomial logistic regression evaluated the associations between LCMM-derived alcohol trajectories and future dementia diagnosis, while controlling for demographic characteristics (age at diagnosis, race, ethnicity, and education). The 3-class model fit best. Classes were distinguished by initial alcohol use, and all classes decreased alcohol consumption over time but to varying degrees (“high initial use with declines that then plateau”, “very high initial use with later decreased use”, and “average users who decreased more with time”). Significant alcohol trajectories by age interactions emerged: Compared to average users, the “high initial users” (OR = 1.03, 95% CI [1.01 - 1.04) and “very high initial users” (OR = 1.39, 95% CI [1.36 - 1.42]) groups showed increased dementia risk with increasing age. No other results were significant. Our findings suggest that early alcohol interventions may be most beneficial in reducing future dementia risk.

